# The effect of career calling on medicine students’ learning engagement: chain mediation roles of career decision self-efficacy and career adaptability

**DOI:** 10.3389/fmed.2024.1418879

**Published:** 2024-11-27

**Authors:** Jingyuan Chen, Hongfeng Liu, Jinku Wang

**Affiliations:** ^1^Information Technology Office, Zhengzhou Railway Vocational & Technical College, Zhengzhou, China; ^2^College of Educational Technology, Northwest Normal University, Lanzhou, China; ^3^College of Nursing, Henan Technical Institute, Zhengzhou, China

**Keywords:** career calling, learning engagement, career adaptability, career decision self-efficacy, medical students in higher vocational college

## Abstract

The study investigates the influence of career calling on the learning engagement of medical students in higher vocational colleges, focusing on the chain mediation roles of career decision self-efficacy (CDSE) and career adaptability. A cross-sectional survey was conducted with 173 students from three vocational colleges across China, utilizing various scales to measure career calling, learning engagement, career adaptability, and CDSE, and structural equation modeling was employed to analyze the survey data. Chain mediation analysis revealed a significant positive correlation between career calling and learning engagement among medical students. Career adaptability was found to partially mediate the relationship between career calling and learning engagement, while CDSE did not exhibit a significant mediating effect. However, when both career adaptability and CDSE were considered as chained mediators, they had a significant indirect effect on the relationship between career calling and learning. The findings highlight the pivotal significance of cultivating a strong career calling and augmenting career adaptability as strategic approaches to bolster learning engagement among medical students in higher vocational colleges.

## 1 Introduction

With the development of the times, health issues have been increasingly prominent. Medical students in higher vocational colleges play a crucial role in primary health care and health management and their training quality has a significant impact on a country’s health care system. However, higher vocational colleges face many problems in the cultivation process of medical students, such as short academic period, heavy academic tasks and high pressure, lack of initiative and motivation in learning, low degree of professional identity, insufficient interest in learning, lack of learning goals and motivation, strong sense of burnout in learning, low quality of learning, low attendance, which seriously affects the quality of talent cultivation (1–3). To improve the quality of talent cultivation and classroom teaching, the core element lies in motivating students to actively and efficiently engage in learning. Therefore, more and more researchers and educational practitioners are committed to enhancing students’ engagement in learning.

Learning engagement refers to a state in which students demonstrate a sustained, positive affective attachment to learning during learning activities (4). It is regarded as an important indicator for measuring learners’ academic achievement and assessing the quality of education, and has received increasing attention in recent years. Existing studies have demonstrated that learning engagement is plastic (5, 6), influenced not only by external environmental factors such as peer relationships, teacher support, family socioeconomic status, and school policies (7–9), but also by individual-level factors like internal motivation and self-efficacy (10, 11). Career calling, a hot topic in recent positive psychology research, has been defined as an approach to work that is inspired by an external summons and provides a means through which one derives meaning and fulfills prosocial motives. Throughout history, individuals who have achieved immortality in the history of medicine and pharmacology all possess benevolence and expertise. This suggests that the cultivation of professional literacy among medical students should not only encompass professional knowledge and skills, but also emphasize ethical, attitudinal, and motivational aspects. When learners internalize their career calling and beliefs, it can inevitably motivate their learning, enhance their efficiency, and promote the advancement of their professional skills. Therefore, for medical students, career calling is a prominent construct. From this perspective, this study focuses on career calling to examine its impact on medical students’ learning engagement.

Several studies have shown that career calling positively predicts preservice teachers’ learning engagement (12, 13). However, there is a dearth of research exploring how and when career calling predicts medical students’ experience of calling toward their careers, which is the objective of the present study. Based on this purpose, the study will distribute questionnaires to medical students in higher vocational colleges located in a certain province in central China and collect relevant data, aiming to gain a deeper understanding of the impact mechanism of career calling on the learning engagement of students in higher vocational colleges. Through this research method, we expect to reveal how career calling influences students’ learning engagement through mediating variables within the framework of the Social Cognitive Career Theory (SCCT), thereby providing empirical evidence for enhancing the learning engagement of medical students and helping them achieve better academic performance.

## 2 Theoretical background and study hypotheses

### 2.1 Career calling and learning engagement

In 2009, Dik and Duffy (14) argue that career calling is a transcendent summons, experienced as originating beyond the self, to approach a particular life role in a manner oriented toward demonstrating or deriving a sense of purpose or meaningfulness and that holds other-oriented values and goals as primary sources of motivation. Bunderson and Thompson (15) point out that career calling is not a dichotomous variable (“yes” or “no”), but rather a continuum, with levels of career calling varying over time. Calling can be categorized into three dimensions: searching for calling, perceiving a calling, and living a calling (16). Searching for calling involves the conscious exploration of career calling by individuals (17). Individuals constantly exploring their own calling to varying degrees throughout their lifelong development; thus, searching for calling is not a once-and-for-all process, but rather a continually cycling one. Perceiving a calling refers to the subjective feeling an individual experiences at the present that he or she wants to engage in a certain kind of work, which not only satisfies the individual’s material needs but also has the social value of the individual’s preference (18). Living a calling refers to the extent to which individuals practice their own career calling (19). Logically speaking, searching for calling, perceiving a calling, and living a calling should occur in sequence, but these three states are often intertwined and exist simultaneously (20). For college students, there are not many opportunities for living a calling because they have not formally started working, so this study mainly focuses on the dimensions of searching for calling and perceiving a calling.

Numerous studies have demonstrated the direct impact of career calling on learning engagement. For example. Chen and Zhang (21) conducted research on nursing students in higher vocational colleges and found that career calling had a significant direct positive effect on their learning engagement. Similarly, Shang et al. (13) found that career calling also had a positive effect on learning engagement through a survey of 1,029 teacher training students at a university in China. When students have a clear sense of career calling, they are more likely to experience the meaning of life, clarify their own personal growth, make correct career plans, and become more actively engaged in their professional studies (22). Similar to goals, career calling serves as a motivational force that encourages students to increase their level of learning engagement and acquire professional knowledge and skills to prepare for their future careers. Therefore, in this study, we propose hypothesis 1: career calling has a positive effect on learning engagement.

### 2.2 Career decision self-efficacy as a mediator

Career Decision Self-Efficacy (CDSE), which refers to individuals’ beliefs or judgments about their ability to achieve career decision-making behavior goals, profoundly affects a range of behaviors in their career development (23). It is considered a key factor that impacts individuals’ career choices and performance (24). According to the SCCT, CDSE helps individuals develop and realize their career self-concepts within their professional roles, thereby influencing the process of career construction. Studies have shown that individuals with high CDSE tend to be more proactive in preparing for their careers and persist in their career pursuits (25); whereas those with low CDSE often hesitate in making career choices (26).

Career calling is a major predictor of CDSE; students with the career calling are more likely to make career-related decisions due to its guidance (27). For instance, Firsick (28) found a direct positive correlation between career calling and CDSE among 433 students. Similarly, Domene (29) conducted a survey among 855 Canadian undergraduate students and found that CDSE fully mediated the impact of career calling on future career expectations, indicating a positive relationship between the two. Duffy and Dik (30) further pointed out that college students with the career calling tend to make more planned, confident, and decisive career decisions, leading to more positive career outcomes. These findings support the positive impact of career calling on CDSE.

SCCT posits that CDSE can shape experiences related to career interests, goal choice, and performance. Extensive empirical research have established that CDSE significantly impacts both career-related variables and life-related factors or instance, CDSE has been found to correlate positively with career outcome expectations (29), intrinsic motivation (31), career exploration behavior (32), career commitment (33), career adaptability (34), the meaning in life and life satisfaction (35). CDSE, which is the capability of individuals to establish and pursue their own personally valued goals during their professional careers (36), often encourages learners with a high level of this ability to engage in learning activities. This is because learning typically provides individuals with more opportunities to acquire knowledge and information related to their professions, emotional support and encouragement, as well as alternative learning experiences through observing and imitating the performance of others in their occupations. All of these factors contribute to the development of abilities that are conducive to the achievement of their professional goals. Given these mixed findings, examining the impact of CDSE on students’ learning engagement is of critical importance. Hence, we propose Hypothesis 2: CDSE mediates the positive impact of career calling on learning engagement.

### 2.3 Career adaptability as a mediator

Career adaptability is one of the core concepts of career construct theory, which describes an individual’s psychological resources when coping with major problems such as vocational tasks, occupational transitions, or unexpected challenges. Comprising four dimensions - attention, control, curiosity, and confidence - it echoes the profound inquiries of ‘Do I have a future?’, ‘Who holds the reins to my future?’, ‘What aspirations do I harbor for the future?’, and ‘Am I capable of fulfilling them?’ (37). Both anticipated and unforeseen vocational tasks serve to catalyze job seekers’ career adaptability, elevating it to a crucial psychological resource that aids in securing employment of superior quality. Some studies have shown that career adaptability is an important factor in an individual’s occupational success and has a significant impact on employment (38) and personal career aspirations (39). College students, prior to their entry into the professional sphere, are prone to encountering setbacks and failures in their career pursuits, with career adaptability emerging as one of the most potent forces in shaping their career successes.

Career preparation and vocational identity are crucial tasks for individuals to undertake at the college level. Individuals constantly adjust their behaviors, assessments, and perceptions of selectivity in accordance with their career goals. Some studies have shown that career adaptability is not only significantly positively related to job search performance, but also impacts students’ academic development (53). It has been shown that career adaptability can mitigate students’ anxieties during the transition from school to work and help students develop the confidence needed to deal with difficult and complex career-related challenges (40).

Douglass and Duffy (27) argued that career adaptability is an important outcome of career calling, because by concern, people can be prepared to gain a sense of purpose in their future careers; control helps to maintain their career calling; curiosity drives them to explore career opportunities they are called to, and confidence provides them with the psychological resources necessary to achieve their career calling. Praskova et al. (41) utilized the calling model of psychological career success to conduct a longitudinal study of 216 Australian students over a six-month period, highlighting that career adaptability is a key competency for career success and an important marker of young people’s development, strongly associated with career calling. Meanwhile, Yang and Chen (42) found that career calling has a significant positive impact on career adaptability through a study of 14,368 nurses in five hospitals in China. Therefore, we believe that career calling provides students with the motivation to achieve their future aspirations, while career adaptability equips them with psychosocial resources that increase the likelihood of adopting the necessary behaviors to achieve desired career outcomes. Based on this, we propose Hypothesis 3: career adaptability plays a positive role in career calling and learning engagement.

In conclusion, career adaptability and CDSE are crucial links between career calling and learning engagement. The Social Cognitive Career Theory (SCCT) proposes that career adaptability serves as a resource for career adjustment, possessing a certain degree of stability. Both individual traits and external environments can influence it. This implies that individuals’ confidence in aspects related to their career decisions affects their adaptability across different career stages. Hou et al. (54) conducted a study with a sample of 810 Chinese college students and found that CDSE mediated the relationship between proactive personality and career adaptability. A study on Korean engineering students revealed that among predictors such as mastery goal orientation, career development support, career decision-making self-efficacy, and interest, CDSE had the strongest predictive effect on career adaptability (43). Therefore, we believe that students with a strong sense of career calling, coupled with high levels of career adaptability and CDSE, contribute to their learning engagement in college. We thus propose Hypothesis 4: Career calling affects learning engagement through the sequential mediation of career adaptability and CDSE.

**Figure 1:**
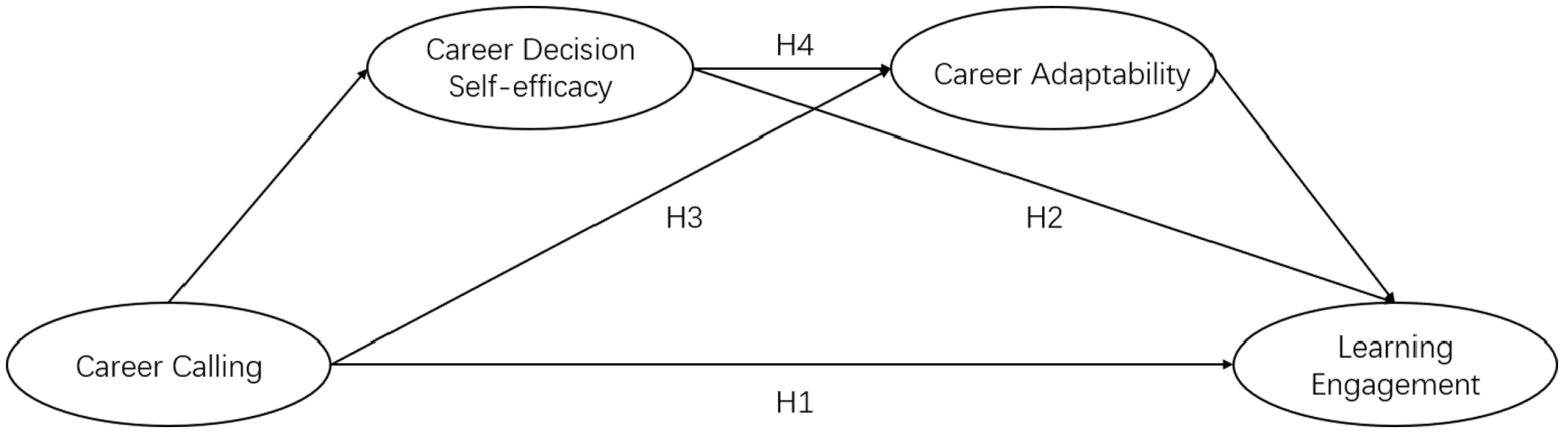
Hypothesized model of mediated relationships between career calling and learning engagement.

Existing research and the SCCT suggest that CDSE and career adaptability are influenced by career calling and, in turn, impact learning engagement. Based on this, the study aims to provide a theoretical framework that can be utilized to enhance medical students’ learning engagement and, in turn, improve the overall quality of medical education and teaching (Figure 1).

## 3 Materials and methods

### 3.1 Participants and procedure

The data used were collected through a survey on the learning engagement of medical students conducted between March and May 2023 at three higher vocational colleges in Henan Province, China. The survey encompassed multiple domains, including learning engagement, career calling, career adaptability, and CDSE, aligning with the research objectives. The survey was distributed to the respondents via WeChat or email with a link to an online questionnaire (Wen Juanxing). Prior to commencing the survey, informed consent was obtained from both the school administrators and the participating students. A researcher provided the respondents with detailed instructions on how to complete the questionnaire, ensuring that the students completed it independently on their personal devices. All responses were anonymized and stored securely for access by the research team for the purpose of this study only.

The survey respondents (*N* = 185) were medical students from three higher vocational colleges in Henan Province, China. Twelve questionnaires were excluded because the answer time was too short (less than 2 min) or the similarity of over 80% of the answers, resulting in 173 valid questionnaires. These valid questionnaires represent a validity rate of 93.51%. The sample comprised 36 male (20.8%) and 137 female (79.2%) participants. Freshmen, sophomores, juniors, seniors accounted for 17.9, 45.7, 35.3, and 1.2% of the participants, respectively. Additionally, 83.8% of the students hailed from rural areas, 14.5% were from small and medium-sized cities, and 1.7% were from large cities.

### 3.2 Measures

(1) The scale of learning engagement

In this study, the Chinese version of the Utrecht Work Engagement Scale for Students (44) was utilized to measure learning engagement. The scale encompasses three domains: vigor (measured by 6 items), dedication (measured by 5 items), and absorption (measured by 6 items), for a total of 17 items. Participants provided ratings on a 5-point scale, with higher scores indicating higher levels of learning engagement. The Cronbach’s alpha coefficients for the scales in this study were 0.899.

(2) The scale of career calling

In this study, a brief version of the CAREER CALLING scale was utilized to assess learners’ career calling, utilizing two question items (27, 45). The scale was titled “I have a sense of purpose in nursing” and “I am very aware of the sense of purpose that nursing should have.” A 5-point Likert scale was employed, with higher total scores indicating higher levels of career calling. The Cronbach’s alpha coefficient for this study were 0.968.

(3) The scale of career adaptability

In this study, the Chinese version of the Career Adaptability Scale was utilized to assess four dimensions of career adaptability: career concordance, career control, career curiosity, and career confidence. Each dimension was comprised of six questions, for a total of 24 questions. Participants provided ratings on a 5-point scale, with higher scores indicating higher levels of career adaptability. The Cronbach’s alpha coefficients for the scales in this study were 0.984.

(4) The scale of CDSE

This study utilized the 25-item short form of the Career Decision Self-Efficacy Scale (CDSE-SF), which was originally developed by Betz et al. (46). The scale was translated and revised by Zhao (47) and is divided into a total of five dimensions, with each dimension consisting of five questions. Participants rated each entry on a 5-point scale, with higher ratings indicating higher levels of career decision self-efficacy. The Cronbach’s alpha coefficients for the scales in this study were 0.990.

### 3.3 Data analysis

This study mainly uses SPSS26.0 as data processing tools. The processing of data encompasses three primary components: descriptive statistics of the survey samples are conducted through SPSS26.0 to examine the basic distribution of the samples. Second, PROCESS3.3, an SPSS macro program developed by Hayes, is utilized to test the mediating effects between variables. Subsequently, the bias-corrected percentile Bootstrap method is employed to generate 5,000 Bootstrap samples to estimate the 95% confidence intervals for the mediating effects. The confidence intervals are determined by examining whether they contain 0; if they do not, it is inferred that the mediating effects are significant.

## 4 Results

### 4.1 Common method bias test

To address common method bias in this study, Harman’s single-factor test will be employed. An exploratory factor analysis of the 68 questions was conducted, and the results indicate that the first common factor accounted for 30.47% of the variance, which is below the critical criterion of 40%. Therefore, there is no significant common method bias in this study.

### 4.2 Descriptive analysis and correlation analysis

The results of the descriptive and correlation analyses (Table 1) indicate that career calling is significantly and positively correlated with learning engagement (*r* = 0. 564, *p* < 0.01), career adaptability (*r* = 0. 466, *p* < 0.01), and CDSE (*r* = 0. 452, *p* < 0.01). Similarly, career adaptability is significantly and positively correlated with learning engagement (*r* = 0.602, *p* < 0.01) and CDSE (*r* = 0.593, *p* < 0.01). There is also a significant positive correlation between learning engagement and CDSE (*r* = 0.858, *p* < 0.01). All of the variables show a moderate or higher degree of correlation (*r* > 0.3, *p* < 0.01), these results meet the conditions for testing the mediation effect and are suitable for further analysis of mediation effects.

**Table 1:** Means, standard deviations, and correlations.

	Mean	SD	1	2	3	4
1. Career Calling	3.887	0.803	1			
2. Learning Engagement	3.627	0.652	0.564^**^	1		
3. Career Adaptability	3.371	0.755	0.466^**^	0.602^**^	1	
4. CDSE	3.497	0.774	0.452^**^	0.593^**^	0.858^**^	1

*N* = 173, *p < 0.05, **p < 0.01, ***p < 0.001.

### 4.3 Hypothesis testing

According to the correlations between the variables in Table 2 and the statistical prerequisites of the mediating effect, the chain mediation model was constructed to investigate the mechanisms of the roles of career adaptability and CDSE in career calling and learning engagement (48). In this study, the chain mediation model was constructed with career calling as the independent variable, learning engagement as the dependent variable, career adaptability, and career self-efficacy as the mediator variables, and gender, type of school attended in high school, and professional intention as the control variables. The 95% unbiased corrected confidence intervals were estimated using the SPSS macro program, PROCESS 3.3, developed by Hayes, and 5,000 Bootstrap samples were drawn using the Bootstrapping method. The model was tested using Model 6 in the PROCESS plug-in.

**Table 2:** Regression analysis of the chain mediating model between career calling and learning engagement.

Variables	Learning engagement		Career adaptability		CDSE	
	*β*	*t*	*β*	*t*	*β*	*t*
Career Calling	0.283	5.514^***^	0.088	3.456^**^	0.435	6.623^***^
CDSE	0.187	1.969	0.806	19.560^***^		
Career Adaptability	0.212	2.171^*^				
R^2^	0.477		0.760		0.204	
F	51.298		269.052		43.861	

*N* = 173, ^*^p < 0.05, ^**^p < 0.01, ^***^p < 0.001.

**Figure 2:**
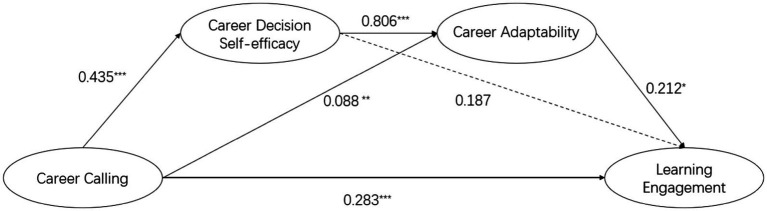
The serial mediation model with career adaptability and CDSE as mediators of the linkage between career calling and learning engagement. ^*^p < 0.05, ^**^p < 0.01, ^***^p < 0.001.

As shown in Table 1, regression analysis indicates that career calling has a significant positive impact on learning engagement (*β* = 0.283, *p* < 0.001), as well as on career adaptability (*β* = 0.088, *p* < 0.01) and CDSE (*β* = 0.435, *p* < 0.001). Career adaptability (*β* = 0.212, *p* < 0.05) significantly positively predicts learning engagement. Additionally, CDSE significantly (*β* = 0.806, *p* < 0.01) positively predicts career adaptability. However, *p* > 0.05, CDSE does not significantly predict learning engagement. The predictive paths of each variable are detailed in Figure 2.

The analysis of mediating effect size (Table 3) indicates that career adaptability and CDSE play significant mediating roles between career calling and learning engagement. The mediating value is 0.174, the 95% confidence interval of [0.068,0.311] that does not contain 0, accounting for 38.07% of the total effect of career calling on learning engagement. Further analysis reveals that three indirect effects are contributing to the mediating effect: indirect effect 1 is the path of Calling -> CDSE -> Learning Engagement, with a mediating effect value of 0.081 and a 95% confidence interval of [−0.018,0.194] that contains 0, thus hypothesis 2 is not supported; indirect effect 2 is the path of Calling -> Career Adaptability -> Learning Engagement, with a mediating effect value of 0.019 and a 95% confidence interval of [0.0001,0.051] that does not contain 0, accounting for 4.16% of the total effect, thus hypothesis 3 is supported; indirect effect 3 is the path of Calling -> Career Adaptability -> Career Decision Self-efficacy -> learning engagement, with a mediating effect value of 0.075 and a 95% confidence interval of [0.006,0.178] that does not contain 0, accounting for 16.41% of the total effect, supporting hypothesis 4.

**Table 3:** Bootstrap analyses of significance of mediation.

Model pathways	Effect	BootSE	BootLLCI	BootULCI	Percentage
Total indirect effect	0.174	0.064	0.068	0.311	38.07%
Ind1: Calling-> CDSE-> Learning Engagement	0.081	0.055	−0.018	0.194	17.72%
Ind2: Calling-> Career Adaptability-> Learning Engagement	0.019	0.013	0.0001	0.051	4.16%
Ind3:Calling-> Career Adaptability-> CDSE-> Learning Engagement	0.075	0.044	0.006	0.178	16.41%

In summary, the above results indicate the existence of indirect effects in the positive impact of career calling on learning engagement. Among these indirect effects, the mediating effect of CDSE is not present, while the mediating role of career adaptability and the chained mediating effect of CDSE and career adaptability are significant.

## 5 Discussion

“How can we enhance the quality of medical students’ education” is a pivotal concern in the realm of medical education reform. Learning engagement, as a key metric of teaching quality, holds immense importance in understanding its influencing factors. Consequently, this study initiates with career calling to delve into its direct impact on medical students’ learning engagement and examines the mediating roles of career adaptability and career decision self-efficacy in the relationship between career calling and learning engagement. The findings indicate that career calling positively predicts learning engagement, with career adaptability acting as a mediator between the two. Furthermore, the chain mediation model reveals that career calling sequentially influences learning engagement through CDSE and career adaptability.

In agreement with the studies by Shang et al. (13) and Yuan and Li (49), this research demonstrates a direct positive relationship between career calling and learning engagement. This finding suggests that medical students who possess a higher sense of career calling tend to engage more actively in their studies, leading to improved academic performance. This correlation can be attributed to the fact that students with a strong career calling perceive their future work as both meaningful and beneficial to society, thus fostering a greater willingness to invest time and effort into their academic pursuits. Therefore, teachers and educational institutions must prioritize the cultivation of career calling among students, enabling them to fully appreciate the significance of their medical profession and ultimately enhance their motivation and engagement in learning.

By validating hypothesis 2, we found that CDSE does not significantly mediate the relationship between career calling and learning engagement. This finding contrasts with the results of Kim (50) but aligns with those of Wu et al. (11). The reasons for this result may be related to the unique nature of medical students’ profession. As medical students are required to address real-life problems, such as diagnosing diseases and providing appropriate treatment plans for patients, they may not derive sufficient self-efficacy solely from academic achievements. Therefore, for medical students, CDSE does not significantly mediate the relationship between career calling and learning engagement. This implies that in the training process of medical students, they should be encouraged to participate in more practical activities and experience a sense of career calling in real task situations, and acquire sufficient CDSE to enhance their level of learning engagement.

By validating hypothesis 3, we have demonstrated that career adaptability serves as a mediator between career calling and learning engagement. In other words, the higher the level of career calling among medical students, the stronger their career adaptability, leading to a higher level of engagement in learning. This finding aligns with previous research (21, 51, 52). Career adaptability, as a psychological construct referring to an individual’s readiness and resources for current or anticipated career development tasks, essentially represents a form of self-regulation that enables individuals to continuously adapt their responses to their environment. This suggests that learners with a high level of career adaptability are more attentive to future trends, well-prepared, proactive in facing changes and challenges in their career, and better able to adapt to changes in their career environment, leading to increased engagement in learning. Therefore, career adaptability, as an important mediating variable, plays a significant role in promoting medical students’ learning engagement. We recommend that schools strengthen career development guidance to enhance students’ career adaptability and ultimately improve their learning engagement.

This study also found that career decision-making self-efficacy and career adaptability serve as chain mediators between career calling and academic engagement. That is to say, career calling is indirectly related to academic engagement through career decision-making self-efficacy and career adaptability. When medical students possess a high level of career calling, it will enhance their CDSE, enabling them to exhibit greater confidence in career decision-making. This increased confidence prompts learners to pay more attention to their future career paths, thus motivating positive behaviors such as taking initiative and trying, ultimately elevating their levels of academic engagement. This study demonstrates that career calling influences medical students’ academic engagement through the independent and combined effects of career adaptability and career decision-making self-efficacy, further exploring the mechanisms underlying the impact of career calling on academic engagement. Understanding the influence of career calling on medical students’ academic engagement levels and the underlying psychological mechanisms is crucial for enhancing their academic performance and teaching quality.

## 6 Limitation and future research

This study has the following limitations, which we hope to improve upon in future research. First, this study obtained cross-sectional data, which does not allow for the determination of causal relationships between these variables. Although the variables used in this study are supported by relevant literature (13, 27, 34, 37), in future research, we will employ longitudinal studies to measure career calling, learning engagement, and other related variables multiple times over a period of one to two years to explore their long-term impacts and dynamic changes. Second, the study sample only included medical students from three high vocational colleges in a central province of China. In future research, we will diversify the study population by including students from different regions, majors, and educational levels to enhance the generalizability and external validity of the research. Third, this study explored the relationship between career calling and learning engagement through the mediating effects of career adaptability and CDSE. However, there are many other factors that influence learning engagement, such as vocational outcome expectations, career identity, family socio-economic status, and so on. In future research, more variables should be considered to obtain more persuasive results and practical recommendations.

## 7 Conclusion

In this study, we explored the relationships among career calling, career adaptability, CDSE, and learning engagement among medical students in higher vocational colleges. The results indicate that career calling has a direct positive impact on learning engagement and that CDSE and career adaptability act as chained mediators between career calling and learning engagement. Specifically, as medical students’ sense of career calling increases, their levels of CDSE and career adaptability also increase, leading to higher levels of learning engagement. The findings of this study not only provide new insights into the relationship between career calling and learning engagement but also suggest possible ways to enhance the learning engagement of medical students.

This study provides implications for enhancing the learning engagement of medical students in higher vocational colleges. Since CDSE and career adaptability play a mediating role between career calling and learning engagement, in the cultivation process of medical students, teachers should not only focus on the transmission of knowledge and skills but also, through practical activities and educational guidance, help students recognize the value and significance of their profession, thereby stimulating their sense of career calling. Additionally, emphasis should be placed on career education to improve their career adaptability, problem-solving skills in career matters, and psychological resilience to better adapt to challenges and changes in their professional careers. Finally, actively providing career counseling and guidance to students, enhancing their CDSE, and boosting their confidence can inspire students’ interest and motivation in learning.

## Data Availability

The datasets presented in this study can be found in online repositories. The names of the repository/repositories and accession number(s) can be found in the article/Supplementary material.

## References

[1] Ikonne U Campbell AM Whelihan KE Bay RC Lewis JH. Exodus from the classroom: student perceptions, lecture capture technology, and the inception of on-demand preclinical medical education. J Osteopath Med. (2018) 118:813–23. doi: 10.7556/jaoa.2018.174, PMID: 30476993

[2] Kanter SL. To be there or not to be there: is attendance really the question? Acad Med. (2012) 87:679. doi: 10.1097/ACM.0b013e31825a5a2a 22643365

[3] White C Bradley E Martindale J Roy P Patel K Yoon M. Why are medical students ‘checking out’ of active learning in a new curriculum? Med Educ. (2014) 48:315–24. doi: 10.1111/medu.12356, PMID: 24528466

[4] Schaufeli WB Salanova M Lez-Roma VG Bakker AB. The measurement of engagement and burnout: A two sample confirmatory factor analytic approach. J Happ Stud. (2002) 3:71–92. doi: 10.1023/A:1015630930326

[5] Appleton JJ Christenson SL Furlong MJ. Student engagement with school: critical conceptual and methodological issues of the construct. Psychol Sch. (2008) 45:369–86. doi: 10.1002/pits.20303

[6] Fredricks JA Blumenfeld PC Paris AH. School engagement: potential of the concept, state of the evidence. Rev Educ Res. (2004) 74:59–109. doi: 10.3102/00346543074001059

[7] Qiu Y Ye P. The influence of family socio-economic status on learning engagement of college students majoring in preschool education: the mediating role of parental autonomy support and the moderating effect of psychological capital. Front Psychol. (2023) 13:1081608. doi: 10.3389/fpsyg.2022.1081608, PMID: 36698604 PMC9868755

[8] Shao Y Kang S. The association between peer relationship and learning engagement among adolescents: the chain mediating roles of self-efficacy and academic resilience. Front Psychol. (2022) 13:938756. doi: 10.3389/fpsyg.2022.938756, PMID: 35992466 PMC9384863

[9] Wolters CA Iaconelli R Peri J Hensley LC Kim M. Improving self-regulated learning and academic engagement: evaluating a college learning to learn course. Learn Individ Differ. (2023) 103:102282. doi: 10.1016/j.lindif.2023.102282

[10] Sökmen Y. The role of self-efficacy in the relationship between the learning environment and student engagement. Educ Stud. (2021) 47:19–37. doi: 10.1080/03055698.2019.1665986

[11] Wu H Li S Zheng J Guo J. Medical students’ motivation and academic performance: the mediating roles of self-efficacy and learning engagement. Med Educ Online. (2020) 25:1742964. doi: 10.1080/10872981.2020.1742964, PMID: 32180537 PMC7144307

[12] Chen HF Xie GB Xin X. The effects of career calling on study engagement: the perspective of social cognitive career theory. J Psychol Sci. (2016) 39:659–65. doi: 10.16719/j.cnki.1671-6981.20160322

[13] Shang W Yu T Liang X Wang J Su J. How does career calling influence preservice teachers’ learning engagement? A multiple mediating roles of occupational self-efficacy and vocational outcome expectation. Front Psychol. (2022) 13:874895. doi: 10.3389/fpsyg.2022.874895, PMID: 35656498 PMC9152323

[14] Dik BJ Duffy RD. Calling and vocation at work: definitions and prospects for research and practice. Couns Psychol. (2009) 37:424–50. doi: 10.1177/0011000008316430

[15] Bunderson JS Thompson JA. The call of the wild: zookeepers, callings, and the double-edged sword of deeply meaningful work. Adm Sci Q. (2009) 54:32–57. doi: 10.2189/asqu.2009.54.1.32

[16] Duffy RD Dik BJ Steger MF. Calling and work-related outcomes: career commitment as a mediator. J Vocat Behav. (2011) 78:210–8. doi: 10.1016/j.jvb.2010.09.013

[17] Duffy RD Sedlacek WE. The presence of and search for a calling: connections to career development. J Vocat Behav. (2007) 70:590–601. doi: 10.1016/j.jvb.2007.03.007

[18] Zhang C Hirschi A Herrmann A Wei J Zhang J. The future work self and calling: the mediational role of life meaning. J Happiness Stud. (2017) 18:977–91. doi: 10.1007/s10902-016-9760-y

[19] Duffy RD Bott EM Allan BA Torrey CL Dik BJ. Perceiving a calling, living a calling, and job satisfaction: testing a moderated, multiple mediator model. J Couns Psychol. (2012) 59:50–9. doi: 10.1037/a0026129 22059426

[20] Dobrow SR. Dynamics of calling: a longitudinal study of musicians. J Organ Behav. (2013) 34:431–52. doi: 10.1002/job.1808

[21] Chen J Zhang X. The impact of career calling on higher vocational nursing students’ learning engagement: the mediating roles of career adaptability and career commitment. Front Psychol. (2023) 14:1111842. doi: 10.3389/fpsyg.2023.1111842, PMID: 37034956 PMC10075228

[22] Duffy RD Douglass RP Autin KL Allan BA. Examining predictors and outcomes of a career calling among undergraduate students. J Vocat Behav. (2014) 85:309–18. doi: 10.1016/j.jvb.2014.08.009

[23] Taylor KM Betz NE. Applications of self-efficacy theory to the understanding and treatment of career indecision. J Vocat Behav. (1983) 22:63–81. doi: 10.1016/0001-8791(83)90006-4

[24] Lent RW Brown SD Hackett G. Toward a unifying social cognitive theory of career and academic interest, choice, and performance. J Vocat Behav. (1994) 45:79–122. doi: 10.1006/jvbe.1994.1027

[25] Bandura A Barbaranelli C Caprara GV. Self-efficacy beliefs as shapers of Children’s aspirations and career trajectories. Child Dev. (2001) 72:187–206. doi: 10.1111/1467-8624.00273 11280478

[26] Hargrove BK Creagh MG Burgess BL. Family interaction patterns as predictors of vocational identity and career decision-making self-efficacy. J Vocat Behav. (2002) 61:185–201. doi: 10.1006/jvbe.2001.1848

[27] Douglass RP Duffy RD. Calling and career adaptability among undergraduate students. J Vocat Behav. (2015) 86:58–65. doi: 10.1016/j.jvb.2014.11.003

[28] Firsick D. The influence of attachment and career calling on career decision self-efficacy, college adjustment, and life satisfaction in undergraduate students. PhD thesis. Greeley, CO: University of Northern Colorado (2016).

[29] Domene JF. Calling and career outcome expectations: the mediating role of self-efficacy. J Career Assess. (2012) 20:281–92. doi: 10.1177/1069072711434413

[30] Duffy RD Dik BJ. Research on calling: what have we learned and where are we going? J Vocat Behav. (2013) 83:428–36. doi: 10.1016/j.jvb.2013.06.006

[31] Shin Y-J. Exploration of the relationship among calling, career decision-making self-efficacy, intrinsic motivation, and career adaptability. Korean J Counsel. (2013) 14:209–26. doi: 10.15703/kjc.14.1.201302.209

[32] Chiesa R Massei F Guglielmi D. Career decision-making self-efficacy change in Italian high school students. J Couns Dev. (2016) 94:210–24. doi: 10.1002/jcad.12077

[33] Chung YB. Career decision-making self-efficacy and career commitment: gender and ethnic differences among college students. J Career Dev. (2002) 28:277–84. doi: 10.1177/089484530202800404

[34] Hamzah SR Kai Le K Musa SNS. The mediating role of career decision self-efficacy on the relationship of career emotional intelligence and self-esteem with career adaptability among university students. Int J Adolesc Youth. (2021) 26:83–93. doi: 10.1080/02673843.2021.1886952

[35] Sari SV. Attaining career decision self-efficacy in life: roles of the meaning in life and the life satisfaction. Curr Psychol. (2019) 38:1245–52. doi: 10.1007/s12144-017-9672-y

[36] Shin JY Steger MF Lee K-H. Major incongruence and career development among American and south Korean college students. J Career Assess. (2014) 22:433–50. doi: 10.1177/1069072713498485

[37] Savickas ML Porfeli EJ. Career adapt-abilities scale: construction, reliability, and measurement equivalence across 13 countries. J Vocat Behav. (2012) 80:661–73. doi: 10.1016/j.jvb.2012.01.011

[38] Fugate M Kinicki AJ Ashforth BE. Employability: a psycho-social construct, its dimensions, and applications. J Vocat Behav. (2004) 65:14–38. doi: 10.1016/j.jvb.2003.10.005

[39] Huang Y Zhao JF Zhang T Zhang Y. Influence of college students’ proactive personality, core self-evaluation, and career adaptability on career expections. Chin Nurs Res. (2022) 36:4404–8.

[40] Hirschi A Vondracek FW. Adaptation of career goals to self and opportunities in early adolescence. J Vocat Behav. (2009) 75:120–8. doi: 10.1016/j.jvb.2009.05.005

[41] Praskova A Hood M Creed PA. Testing a calling model of psychological career success in Australian young adults: a longitudinal study. J Vocat Behav. (2014) 85:125–35. doi: 10.1016/j.jvb.2014.04.004

[42] Yang C Chen A. The double-edged sword effects of career calling on occupational embeddedness: mediating roles of work–family conflict and career adaptability. Asian Nurs Res. (2020) 14:338–44. doi: 10.1016/j.anr.2020.09.005 33049413

[43] Park S Park SY. Career adaptability of South Korean engineering students: personal and contextual influencing factors. European J Train Dev. (2020) 44:469–88. doi: 10.1108/EJTD-10-2019-0181

[44] Gan Y Yang M Zhou Y Zhang Y. The two-factor structure of future-oriented coping and its mediating role in student engagement. Personal Individ Differ. (2007) 43:851–63. doi: 10.1016/j.paid.2007.02.009

[45] Dik BJ Eldridge BM Steger MF Duffy RD. Development and validation of the calling and vocation questionnaire (CVQ) and brief calling scale (BCS). J Career Assess. (2012) 20:242–63. doi: 10.1177/1069072711434410

[46] Betz NE Hammond MS Multon KD. Reliability and validity of five-level response continua for the career decision self-efficacy scale. J Career Assess. (2005) 13:131–49. doi: 10.1177/1069072704273123

[47] Zhao FX. A study on the compilation and application of career decision-making scale for college students. PhD thesis. Hangzhou: University of Zhejiang (2005).

[48] Wen Z Ye B. Analyses of mediating effects: the development of methods and models. Adv Psychol Sci. (2014) 22:731–45. doi: 10.3724/SP.J.1042.2014.00731

[49] Yuan L Li L. Influence of ethnic minority normal students’ career calling on their learning engagment——an analysis based on mediating effect of psychology capital. J Tibet Univ. (2021) 36:226–34. doi: 10.16249/j.cnki.1005-5738.2021.04.029

[50] Kim J Doo MY. The effects of motivation, career decision-making self-efficacy, and self-regulation on learning engagement of junior college students. J Coll Stud Dev. (2022) 63:432–48. doi: 10.1353/csd.2022.0036

[51] Guo Y Guan Y Yang X Xu J Zhou X She Z. Career adaptability, calling and the professional competence of social work students in China: a career construction perspective. J Vocat Behav. (2014) 85:394–402. doi: 10.1016/j.jvb.2014.09.001

[52] Yu HB Li XW. The influence of career adaptability on academic performance and job search performance of students in free teacher education programs: the moderating of career identity. Chinese J Spec Educ. (2015):75–80.

[53] Capela AF Oliveira I. Career adaptability and Students’engagement in higher education, ICERI2023 proceedings. IATED. (2023):835–42. doi: 10.21125/iceri.2023.0291

[54] Hou C Wu L Liu Z. Effect of proactive personality and decision-making self-efficacy on career adaptability among Chinese graduates. Soc Behav Pers. (2014) 42:903–912. doi: 10.2224/sbp.2014.42.6.903

