# Supplementary material for: The effect of career calling on medicine students’ learning engagement: chain mediation roles of career decision self-efficacy and career adaptability
Source: Front Med (Lausanne). 2024 Nov 27;11:1418879. doi: 10.3389/fmed.2024.1418879 (PMC11631594; doi:10.3389/fmed.2024.1418879)
Supplement: Supplementary file 1 [file Table_1.DOCX]

**The questionnaire of medical students’ career calling and learning engagement**

Instructions: Thank you for taking part in this survey. The aim of this anonymous survey is to gain an understanding of medical students' career calling and learning engagement, as well as the main influencing factors. Your responses will only be used for statistical analysis and will remain confidential. It is essential that you answer truthfully and accurately in order to ensure the accuracy of our study.

1. Your gender:

A. Male B. Female

2. What is your major?

A. Traditional Chinese Pharmacy

B. Pharmacy

C. Food and Drug Administration

D. Pharmaceutical Business and Management E. Others

3. Your Grade:

A. Freshman B. Sophomore C. Junior

4. Where are you come from?

A. Rural towns

B. Small and medium-sized cities

C. Big cities

5. Which categories of high school do you attend

A. Secondary Vocational and Technical College

B. Ordinary High School

C. Key High School

6. Is college application what you want it to be?

A. Yes B. No

7. What is the highest level of education your father has achieved?

A. Primary school and below

B. Secondary or technical secondary school

C. Higher Vocational School

D. undergraduate and above

8. What is the highest level of education your mother has achieved?

A. Primary school and below

B. Secondary or technical secondary school

C. Higher Vocational School

D. undergraduate and above

Part Two: Calling Career Scale

Instruction: Read each item and compare it to your feelings; then select the level that corresponds to your level of conformity from the checkboxes below.

| Question | 1. complete disagreement | 2. disagree | 3. uncertainty | 4. agree | 5. complete agreement | |
| --- | --- | --- | --- | --- | --- | --- |
| 1. I have a calling to the career I am going to engage in. | 1 | 2 | 3 | 4 | 5 |  |
| 2. I have a good understanding of my calling as it applies to my career | 1 | 2 | 3 | 4 | 5 |  |

Part Three: learning engagement

Instruction: Read each item and compare it to your feelings; then select the level that corresponds to your level of conformity from the checkboxes below.

| Question | 1. completely disagreement | 2. disagree | 3. uncertainty | 4. agree | 5. completely agreement |
| --- | --- | --- | --- | --- | --- |
| 1. As soon as I wake up in the morning, I am happy to go and study | 1 | 2 | 3 | 4 | 5 |
| 2. I feel energized when I study | 1 | 2 | 3 | 4 | 5 |
| 3. Even when learning doesn’t go well, I don't get discouraged and I persevere | 1 | 2 | 3 | 4 | 5 |
| 4. I can study for a long time without taking a break | 1 | 2 | 3 | 4 | 5 |
| 5. I can recover quickly from mental fatigue when studying | 1 | 2 | 3 | 4 | 5 |
| 6. I feel strong and motivated when I am studying | 1 | 2 | 3 | 4 | 5 |
| 7. I find learning challenging | 1 | 2 | 3 | 4 | 5 |
| 8. Learning inspires me | 1 | 2 | 3 | 4 | 5 |
| 9. I am passionate about learning | 1 | 2 | 3 | 4 | 5 |
| 10. I am proud of my learning | 1 | 2 | 3 | 4 | 5 |
| 11. I find that my learning is purposeful and meaningful | 1 | 2 | 3 | 4 | 5 |
| 12. When I learn, I forget everything around me | 1 | 2 | 3 | 4 | 5 |
| 13. When I study, I feel that time flies | 1 | 2 | 3 | 4 | 5 |
| 14. When I study, all I can think about is my studies | 1 | 2 | 3 | 4 | 5 |
| 15. I find it hard to let go of my studies | 1 | 2 | 3 | 4 | 5 |
| 16. I am immersed in my studies | 1 | 2 | 3 | 4 | 5 |
| 17. I feel happy when I am fully engaged in my studies | 1 | 2 | 3 | 4 | 5 |

Part Four: career adaptability scale

Instruction: Read each item and compare it to your feelings; then select the level that corresponds to your level of conformity from the checkboxes below.

| Question | 1. completely disagreement | | 2. disagree | 3. certainty | 4. agree | | 5. completely agreement |
| --- | --- | --- | --- | --- | --- | --- | --- |
| 1.Thinking about what my future will be like | | 1 | 2 | 3 | 4 | 5 | |
| 2. Realizing that today's choices shape my future | | 1 | 2 | 3 | 4 | 5 | |
| 3. Preparing for the future | | 1 | 2 | 3 | 4 | 5 | |
| 4. Becoming aware of the educational and career choices that I must make | | 1 | 2 | 3 | 4 | 5 | |
| 5. Planning how to achieve my goals | | 1 | 2 | 3 | 4 | 5 | |
| 6. Concerned about my career | | 1 | 2 | 3 | 4 | 5 | |
| 7.Keeping upbeat | | 1 | 2 | 3 | 4 | 5 | |
| 8. Making decisions by myself | | 1 | 2 | 3 | 4 | 5 | |
| 9. Take responsibility for my actions | | 1 | 2 | 3 | 4 | 5 | |
| 10. Sticking up for my beliefs | | 1 | 2 | 3 | 4 | 5 | |
| 11. Counting on myself | | 1 | 2 | 3 | 4 | 5 | |
| 12. Doing what's right for me | | 1 | 2 | 3 | 4 | 5 | |
| 13. Explore my surroundings | | 1 | 2 | 3 | 4 | 5 | |
| 14. Look for opportunities to grow as a person | | 1 | 2 | 3 | 4 | 5 | |
| 15. Investigating possible opportunities before making a choice | | 1 | 2 | 3 | 4 | 5 | |
| 16. Observe different ways of doing things | | 1 | 2 | 3 | 4 | 5 | |
| 17. Probing deeply into questions I have | | 1 | 2 | 3 | 4 | 5 | |
| 18. Being curious about new opportunities | | 1 | 2 | 3 | 4 | 5 | |
| 19. Performing tasks efficiently | | 1 | 2 | 3 | 4 | 5 | |
| 20. Taking care to do things well | | 1 | 2 | 3 | 4 | 5 | |
| 21. Learn new skills | | 1 | 2 | 3 | 4 | 5 | |
| 22. Working up to my ability | | 1 | 2 | 3 | 4 | 5 | |
| 23. Overcomes obstacles | | 1 | 2 | 3 | 4 | 5 | |
| 24. Solve problems | | 1 | 2 | 3 | 4 | 5 | |

Part Five: CDSE Scale

Instruction: Read each item and compare it to your feelings; then select the level that corresponds to your level of conformity from the checkboxes below.

| Question | 1. completely disagreement | | 2. disagree | 3. certainty | 4. agree | | 5. completely agreement |
| --- | --- | --- | --- | --- | --- | --- | --- |
| 1. Use the Internet to find career information that interests you | | 1 | 2 | 3 | 4 | 5 | |
| 2. Choose a career from the ones you are considering | | 1 | 2 | 3 | 4 | 5 | |
| 3. Make plans for your career goals in the next 5 years | | 1 | 2 | 3 | 4 | 5 | |
| 4. Identify action steps to address learning difficulties encountered in the selected major | | 1 | 2 | 3 | 4 | 5 | |
| 5. Accurately evaluate your own abilities | | 1 | 2 | 3 | 4 | 5 | |
| 6. Choose a career from the ones you are considering | | 1 | 2 | 3 | 4 | 5 | |
| 7. Determine the action steps to be taken to facilitate the successful completion of the course | | 1 | 2 | 3 | 4 | 5 | |
| 8. When faced with setbacks, remain committed to your professional or career goals | | 1 | 2 | 3 | 4 | 5 | |
| 9. Determine what your ideal job is | | 1 | 2 | 3 | 4 | 5 | |
| 10. Identify the employment trend of a certain occupation in the next 10 years | | 1 | 2 | 3 | 4 | 5 | |
| 11. Choose a career that matches the lifestyle you aspire to | | 1 | 2 | 3 | 4 | 5 | |
| 12. Prepare a good resume for job hunting | | 1 | 2 | 3 | 4 | 5 | |
| 13. When you don't like your preferred major, change your major | | 1 | 2 | 3 | 4 | 5 | |
| 14. Know what you value most in a career | | 1 | 2 | 3 | 4 | 5 | |
| 15. Ascertain the average annual income of employees in a certain occupation | | 1 | 2 | 3 | 4 | 5 | |
| 16. When making career decisions, you will not worry about whether it is right or wrong | | 1 | 2 | 3 | 4 | 5 | |
| 17. If you are not satisfied with your chosen career, you can change your career | | 1 | 2 | 3 | 4 | 5 | |
| 18. To achieve career goals, know what you are willing to sacrifice | | 1 | 2 | 3 | 4 | 5 | |
| 19. Talk with people who are already working in their field of interest | | 1 | 2 | 3 | 4 | 5 | |
| 20. Choose a job or career that fits your interests | | 1 | 2 | 3 | 4 | 5 | |
| 21. Identify employers, businesses, or institutions that may be relevant to your career | | 1 | 2 | 3 | 4 | 5 | |
| 22. Know what kind of lifestyle you want | | 1 | 2 | 3 | 4 | 5 | |
| 23. Find information about enrollment in professional colleges | | 1 | 2 | 3 | 4 | 5 | |
| 24. Successfully deal with the job interview process | | 1 | 2 | 3 | 4 | 5 | |
| 25. When unable to obtain the preferred major or career, identify reasonable alternatives | | 1 | 2 | 3 | 4 | 5 | |
